# Investigating the impact of adventure education on children’s physical, cognitive and socio-emotional development: A mixed method systematic review

**DOI:** 10.1371/journal.pone.0327181

**Published:** 2025-06-30

**Authors:** Ruhina Binta A. Ghani, Patrick W. C. Lau, Nike Lu, Peng Zhou, Jing Jing Wang

**Affiliations:** 1 Department of Sports and Health Sciences, Academy of Wellness and Human Development, Faculty of Arts and Social Sciences, Hong Kong Baptist University; 2 Laboratory of Exercise Science and Health, Beijing Normal-Hong Kong Baptist University, Zhuhai, China; 3 Mass Sports Research Centre, China Institute of Sport Science, Beijing, China; University of Lahore - Raiwind Road Campus: The University of Lahore, PAKISTAN

## Abstract

This mixed-method systematic review examines how Adventure Education is perceived by children and its impact on their development. Children’s age ranged between 06–18 years. AE been reported to improve participants’ leadership, self-efficacy, peer support, relationship skills, independence, and the ability to follow instructions. Six quantitative research studies have shown a positive association of AE with various socio-emotional development outcomes. Participants exhibited enhanced resilience, hedonic balance, and life satisfaction. Individuals engaged in AE were found to experience reduced mental health issues and stress levels. One study found that the AE program significantly impacted physical activity outcomes, with effect sizes ranging from 0.029 to 0.663. However, long-term effects could not be definitively concluded due to the lack of follow-up measures of the interventions.

## Introduction

It is indisputable that regular physical activity (PA) has positive health effects, and almost everyone can reap these benefits [1,2]. The body of research demonstrating the value of an active lifestyle for optimum health and well-being has increased dramatically. Engaging in regular PA, i.e. any movement that requires energy and can be done during leisure, transportation, or work [3], has a multitude of health benefits, including the prevention and management of noncommunicable diseases like cardiovascular diseases, cancer, and diabetes [4]. It has been found to alleviate symptoms of depression and anxiety, improve cognitive abilities, and promote healthy development in young individuals, all of which contribute to overall well-being [5]. In children and adolescents, regular PA enhances physical fitness [6], cardiometabolic health [7–9], bone health [10], cognitive outcomes [11–15], mental health [16–18] and reduces adiposity [19,20]. Children aged between 5 to 17 years should engage in 60 minutes of PA per day, as WHO [21] recommends. It suggests that moderate to vigorous aerobic activities and exercises that enhance muscular and bone strength should be done over the week. One should restrict the duration of sedentary behaviour, especially the time spent engaging in recreational screen activities [21].

In the 21st century, a lifestyle shift has caused individuals to spend more time indoors and less time engaging with nature. Researchers have discovered that children are spending limited time outdoors due to the lack of suitable outdoor environments, concerns about their safety, and extended hours devoted to formal education. [22]. Currently, the amount of time children spend outside is lower than the duration their parents spend outdoors during their childhood [23]. Challenging children physically and mentally through adventure education (AE) positively influences their PA, cognition and socio-emotional functions [1,2]. The foundation of AE centres around the belief that direct experiences are instrumental in learning about oneself, others, and the world [24,25]. This approach involves participating in PA that encourage collaboration, promote moral action and help to develop self-esteem, communication skills, and problem-solving abilities [26–29]. Students must react to and embrace difficulty and act spontaneously in a creative, new learning environment that encourages exploration and unpredictability [30,31]. AE intervention significantly enhances critical thinking [32], group belonging [33–35], resiliency [36,37], academic efficacy [38], and pro-social behaviour [39] in children. It also reduces school truancy [40] and improves subjective well-being [41,42].

In confined natural environments, problem-solving in a team and effective collaboration, AE programs effectively cultivate social competencies in adolescents, particularly in situations that require assertiveness through the integration of PA [43]. These social skills are essential for evaluating job candidates, and their absence can result in marginalisation or social exclusion. [24]. Whereas, AE is an intervention that can help adolescents in their overall development. It can teach them important life skills and values while promoting interdependence and competency [44]. AE can also be a powerful tool for building confidence and resilience [45,46]. Participants in AE programs often exhibit higher levels of self-esteem and suffer from less depression [47]. The AE program promotes self-understanding, interpersonal learning, socialising techniques, and cohesion. It also encourages altruism, imitative behaviour, communication and a sense of universality [48].

This study aspires to expand the current knowledge on AE by building on the findings of a previous systematic review. The previous review revealed that AE positively impacted the physical development of non-healthy children and was an effective approach for fostering social development in healthy and unhealthy children [49]. This study holds significance as it focuses on the impact of AE on the overall growth and development of healthy children, which is a crucial and underexplored area. Furthermore, the study addresses the lack of learner-focused research in AE by prioritising participants’ experiences through qualitative analysis. It bridges the research gap with a mixed-methods approach, combining quantitative and qualitative data. Children from middle childhood (06-18 years) were selected for the review due to their developmental readiness to engage in outdoor experiences that foster independence, social skills, and resilience, marking a significant transition from the home-centred activities of early childhood,

Thus, the objective of this study is twofold:

1. Explore children’s perceptions of AE on their physical, cognitive and social-emotional development.
2. Determine if attending an AE program is associated with physical, cognitive and social-emotional development.

## Methods

A systematic review was conducted to analyse qualitative and quantitative studies exploring the effects of AE on children’s growth and development. The review was registered with the International Prospective Register of Systematic Reviews (CRD42024520033) in March 2024. The Preferred Reporting Items for Systematic Review and Meta-Analyses [50] guidelines were followed for reporting this review.

### Eligibility criteria

The PI(E)COS (Population, Intervention or Exposure, Comparison, Outcomes, and Study Design) framework was used to select articles for this systematic review [51].

#### Population.

Children aged 6 to 18 years were included in this review. The eligibility of participants was assessed based on the mean age, range, or median reported in the study. Children with complete physical, mental, and social well-being and not merely in the absence of disease or infirmity [52], were eligible for the review. Studies that exclusively included children with specific diseases or conditions, such as autism, physical disabilities, attention deficit hyperactivity disorder, mental disorders, etc., were excluded. Also, the study used AE as a therapy was excluded,

#### Intervention or exposure.

Articles describing AE interventions specifically implemented in wilderness or outdoor environments were included. These settings typically comprised natural landscapes such as forests, mountains, rivers, or other outdoor areas, facilitating experiential learning and engagement with nature. AE programs incorporating challenges or activities requiring physical, mental, or emotional engagement from participants were considered. These challenges may have encompassed outdoor activities like hiking, rock climbing, team-building exercises, problem-solving tasks, and other adventure-based experiences.

#### Comparison.

Articles with experimental and control groups and single-group pre- and post-test experiments were included for comparison purposes.

#### Outcomes.

The main outcomes of this review encompassed the social, emotional, cognitive development, and physical well-being of children participating in AE programs.

#### Study design.

This review encompassed all study designs, including randomised controlled trials (RCTs), quasi-experimental studies, observational studies, qualitative studies, and mixed-methods studies.

### Search strategy

In January 2024, a thorough search strategy was implemented to conduct a comprehensive systematic review of peer-reviewed articles. To ensure comprehensive literature coverage, a thorough approach was adopted using seven major databases. The selected databases for the search included Sportdiscuss (EBSCOHost), PubMed, Medline, PsycINFO, EMBASE (Ovid), Web of Science, and ERIC.

### Keywords

The search strategy employed a combination of appropriate keywords and controlled vocabulary terms to maximise the retrieval of relevant articles. The keywords and controlled vocabulary terms were carefully selected based on their relevance to the topic of AE and its impact on children’s development. The search terms included (adventure education OR adventure-based training OR outdoor adventure OR adventure experience OR adventure activity OR experiential learning OR experiential education) AND (Pre-schooler OR Schoolchild OR School-age OR Child OR Paediatric OR Adolescent OR Youngster OR Teen OR Minor OR Youth OR Young person OR Juvenile). Additional filters were applied to refine further the search results, including language (English) and publication date (2000 to the present). These filters ensured that only articles meeting the specified criteria were included in the review.

### Extraction of the study

The retrieved articles were uploaded to EndNote software (version 20; Clarivate) for further selection and to remove duplicates. Following the elimination of duplicates from the search results, the titles underwent an initial screening. One reviewer (RG) reviewed all titles and abstracts, removing studies that were irrelevant to the research question. In the subsequent stage, two reviewers (RG and LN) independently evaluated the titles and abstracts of the remaining citations against the inclusion criteria to identify potentially relevant papers. During the final stage, both researchers (RG and LN) conducted a thorough review of the full articles to determine eligibility based on the predefined criteria. Throughout the process, a consensus regarding inclusion was reached through discussions among all reviewers, with the principal supervisor (PL) participating when necessary to facilitate negotiation [13,8].

### Quality appraisal for selected study

The included studies’ methodological quality and risk of bias were assessed using the Joanna Briggs Institute (JBI) checklist, a widely recognised tool for evaluating study characteristics [53,54]. The researchers utilised the JBI QARI critical appraisal tool to evaluate the potential for bias in each study’s design, methodology, and analysis. This tool includes 10 questions for qualitative studies, 13 for RCTs, and 9 for quasi-experimental studies, allowing for a comprehensive assessment of possible biases. Furthermore, the selected articles were categorised into high, medium, and low quality based on a grading system established by Higginbottom and colleagues [55]. Later, Kabir and Chan [56] also utilised this grading system in their meta-synthesis, assigning scores based on the overall quality of studies.

## Result

### Summary of search strategies

**Fig 1:**
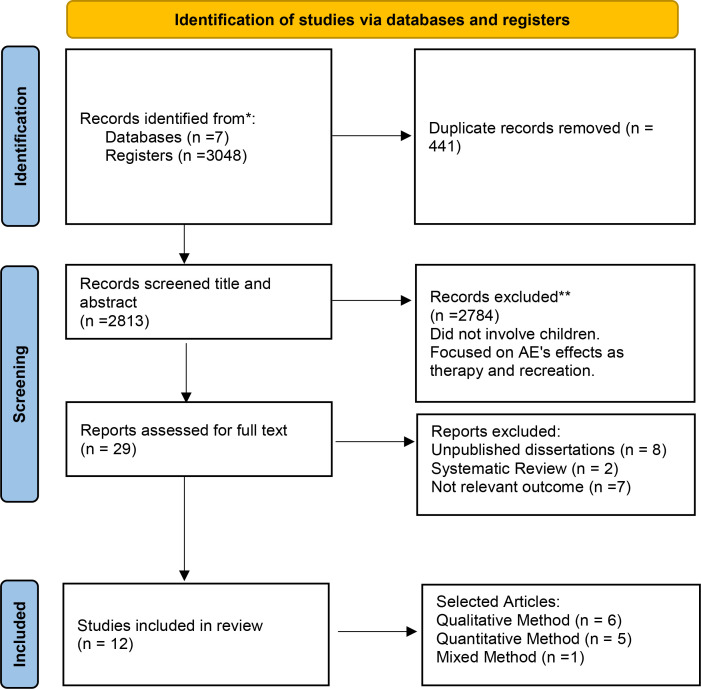
Flow diagram of selected articles following PRISMA.

The search across seven databases using specific keywords yielded 3048 articles. After removing duplicates using EndNote 20 software, 441 articles were removed, resulting in 2813 remaining articles. The primary aim of the review was to explore the effects of AE specifically involving healthy children. Therefore, any studies that did not include healthy child participants were excluded. Additionally, articles that framed AE solely within the context of therapy were also disregarded, as they did not fit the broader scope of the review. These articles were screened based on their title and abstract, excluding 2784 articles as they were deemed irrelevant to the study. Subsequently, the remaining 29 articles underwent a full-text assessment. Among them, 17 articles were excluded due to being unpublished dissertations (8), systematic reviews (2), or lacking relevant outcomes (7). Ultimately, 12 articles (Fig 1) were selected for inclusion in this mixed-method systematic review. Out of the 12 selected articles, 6 used qualitative research methodology, 5 articles were based on quantitative research methodology, and one of the selected articles used mixed-method research.

### Study characteristics and sample size

The selected articles used diverse methodologies. Blaine and Akhurst [57] employed a quasi-experimental, qualitative approach; Zygmont and Naidoo [44] utilised phenomenography;Ingman [58] applied educational connoisseurship and criticism. Chung et al. [47] conducted a single-blind randomised controlled trial (RCT); Mutz et al. [59] implemented a pre-post-test design; Scarf et al. [60] adopted a mixed methods approach; Mutz and Müller [61] implied longitudinal research. Mackenzie et al. [62] explored using repeated measures. Richmond et al. [63], Orson et al. [64], and Down et al. [65] utilised qualitative methodologies. Ritchie et al. [66] adopted the Expedition Ethnography method in their study.

The qualitative study analysed a sample size ranging from 24 to 144 individuals, including male and female participants aged 9 to 19. The selected quantitative studies had a sample size varying from 12 to 228 individuals, with participants aged 12 to 19 years.

### Perception of children participating in AE

In line with objective 1, six qualitative and one mixed-method studies were selected for thematic analysis. Regarding data collection, the studies relied on direct observation, focus group discussions, and individual interviews to capture the perceptions and experiences of the learners participating in the AE programs. The framework by Campel et al. [67] was utilized for thematic analysis. The process starts with generating a research idea suitable for synthesis, then identifying relevant studies that meet the inclusion criteria, and extracting concepts and ideas from the chosen studies. Next, the connections between these studies are explored to generate themes, from which three high-order themes were identified (Table 1).

**Table 1:** Characteristics of selected qualitative studies.

Study and country	Study design	Participant Characteristics	Intervention	Data collection and analysis	Themes	Phenomenon of interest
Blaine and Akhurst (2020) South Africa	Quasi-experimental research design with qualitative inquiry	S: *N = *144 (males = 98, females = 54) Mean age = 16.5 years, SD = 3.45 years)	−21 days -Activities: canoeing, cycling, running, and hiking.	-Focus group semi-structured interview -Thematic analysis	**F**: Friendships **L**: Life Lessons **O**: Out-of-comfort zone **U**: Ubuntu **R**: Reflection **I**: Intrapersonal Development **S**: Social Development **H**: Humility and Gratitude **I**: Individual Differences (including sex) **N**: Nature (a)**G**: Group Dynamics	Explored the psychosocial outcomes of an outdoor adventure program for adolescents
Down et al. (2023) Australia	Qualitative descriptive approach	N = 29 Age: 15-16y	-Outdoor adventure education	-Five focus groups -Thematic analysis	(b)Perceptions of the outdoors, motivators for participation (c)barriers to participation staff traits, and [2] ideal program components	Exploring the perceptions of adolescent preferences regarding program components designed to enhance their well-being during a secondary school outdoor adventure education program.
Ingman (2018) USA	Educational connoisseurship and criticism	N = 41(14 females and 27 males) Age: 9 to 19 years	−11 to 8 days -Activities: backpacking, fishing, participating in low-initiative activities, hiking, swimming, and camping, climbing, rafting, camping, hiking	-Observation, 71 interviews were conducted using semi-structured and mobile or conversational techniques. - Thematic analysis	(a)sensory encounters full attention and [4] aesthetic paradox	Investigating the experiences and interpretations of participants during adventure education [6] activities.
Orson et al. (2020) USA	Qualitative Research	N = 32 (M:16, F:16) Age: 14–18y (*M* = 15.2, *SD* = 1.3)	− 5 to 14 days -Philadelphia Outward Bound School expeditions	- six group interviews with semi-structured open-ended questions - Thematic analysis	(a)Youth learned through struggling with challenges (b)Peers’ support helped students overcome challenges and learn (c)Youth embraced a culture of compassion and commitment	Challenges and Peers’ Contributions in Social-Emotional Learning in Outdoor Adventure Education Programs
Richmond et al. (2018) USA	Qualitative Research	N = 24 (M:0, F:24) Age: 13 to 18 years	− 6 days -Activities: backpacking	−10 semi-structured, open-ended interviews. -Thematic analysis	(a)Social connectedness (b)Self-efficacy in leadership (c)Recalibrated sense of self	The significance of the skills, beliefs, and behaviours that contribute to student achievement within the classroom and in broader contexts is emphasized.
Ritchie et al. (2015) Canada	Expedition Ethnography	N = 43 (M:27, F: 16) Age: 12–18y Mean Age: 14.7 y	−10-days -Activities: camping	-observations, journals and interviews -Thematic analysis	Connecting to the Good Life	Understanding the outdoor adventure leadership experience for promoting resilience and well-being for Indigenous youth.
Zygmont and Naidoo (2017) South Africa	Phenomenography	*N = *37 (M:19, F: 18) Grade 9 high school learners	− 27 days -Activities: walking, bicycling, canoeing and raft building	-Semi-structured interview -Phenomenographic analysis	(a) long gruelling school Hike [4] school initiation/rite of passage programme [2] once-in-a-lifetime group adventure (d) multifaceted learning and development opportunity	Investigate the different methods of wilderness adventure programs and identify variations in program outcomes.

#### Theme 1: Somatic experience.

AE serves as an effective method for enhancing physical well-being in children by integrating PA, such as climbing and hiking, with experiential learning in natural settings, thereby promoting fitness while also improving cardiovascular health, strength, flexibility, and motor skills [68]. Adopting the AE in the educational curriculum can cultivate an interactive atmosphere that promotes lifelong healthy habits and provides strategies for incorporating PA into students’ everyday lives [69]. In the current review, the first theme focuses on how AE influences somatic experience. Involvement in these programs increases children’s awareness of their bodies and enhances their physical involvement.

The physically demanding and engaging nature of adventure-based learning experiences fostered their physical development. One of the participants mentioned,

*“I found that the more spiritual you are, the happier you are emotionally. I don’t want to stereotype or anything, but the more active you are, the happier you are too. It seems like happiness is an emotion, and it lifts your spirits. Once you are happy, you are more active. Once you are active, you are more alert and have a positive outlook on life. That’s the best way I could put it”* [66].

Although AE improves PA among children, participants from five articles [44,58,64,65] also mentioned the physically demanding nature of AE activities. Engaging in challenging physical tasks, such as rock climbing, hiking, and obstacle courses, enhances participants’ strength, endurance, and overall physical fitness. However, the rigorous nature of AE programs can also be taxing, as evidenced by one participant’s comment:

*“My feet hurt so bad. I hurt my ankle the beginning of the second day and that was really hard. I thought to myself that Outward Bound was torture… I felt sad and angry.”* [64].

The evidence strongly suggests that AE is effective in improving physical well-being. Nevertheless, it is crucial to acknowledge that the intense physical nature of the activities can potentially cause discomfort, injury, and negative emotional responses among participants. To address this, meticulous program design and thorough preparation are important to effectively manage the physical challenges in relation to participants’ capabilities and comfort levels.

#### Theme 2: Stimulation for learning.

The findings from these studies suggest that AE can be an effective approach for facilitating the development of crucial self-care and independent living skills [58,64]. By placing participants in situations that require them to adapt, solve problems, and collaborate, AE not only stimulates learning but also equips individuals with transferable skills that are vital for their personal growth and everyday life [70]. Moreover, the life skills acquired through AE are instrumental in shaping responsible adults.

The thematic analysis of seven studies indicates that AE provides participants with opportunities to learn and practice various practical life skills, including cooking, cleaning, and setting up camping equipment such as tents. These activities are often embedded within the structure of AE programs, offering participants hands-on experiences that foster the development of essential skills for independent living. The unfamiliar and often challenging environments encountered during AE activities serve as powerful motivators, encouraging participants to acquire and apply these practical skills in real-time contexts. For instance, one participant noted

*“Like, I can cook for myself. I can pitch my own tent. I can do my own thing you know”* [57].

AE fosters an active learning process where participants engage deeply with difficult challenges, both physical and emotional. These challenges often require participants to push through discomfort, frustration, and uncertainty, cultivating resilience and determination [64]. Successfully navigating these obstacles allows participants to gain valuable reflective insights, facilitating self-discovery and a deeper understanding of their own strengths and abilities. This process not only enhances their confidence but also reinforces their belief in their capacity to overcome future challenges, both within and beyond the context of AE [64].

AE creates immersive environments that demand participants’ full attention and focus, often by placing them in dynamic and unpredictable settings. For example, one observation described a group paddling down a river in complete silence except for the crashing water and muffled instructions from their guides. The intense focus required to navigate such an environment underscores how AE fosters attentiveness by drawing participants into the present moment [58]. These scenarios compel participants to actively engage with their surroundings and respond to immediate challenges, enhancing their ability to concentrate on tasks at hand. The increased sensory engagement and the necessity to process real-time information, such as instructions from instructors or changes in the environment, train participants to remain attentive and alert [58]. This capacity for full attention not only supports success in adventure-based activities but also translates to improved focus and mindfulness in other areas of life, including academic and personal responsibilities.

#### Theme 3: Adventure education as a pathway to improve socio-emotional development.

This theme underscores AE as a potent tool for promoting socio-emotional development, supported by findings from various studies. The analysis reveals several interconnected sub-themes that highlight specific aspects of socio-emotional growth facilitated by AE. These sub-themes include Leadership, which focuses on developing decision-making and guiding abilities; Self-efficacy, reflecting an increased confidence in one’s capabilities; Peer Support, which emphasizes strengthened social bonds and collaboration; Stress Relief, demonstrating the ability to regulate emotions in challenging situations; Relationship Skills, cantered on improved interpersonal communication; Independence, showcasing the capacity to manage tasks autonomously; and Following the Guide, which highlights participants’ trust in and learning from individuals in leadership roles. Together, these sub-themes reinforce the transformative potential of AE in enhancing socio-emotional competencies.

### Leadership

Leadership emerged as a critical outcome of AE participation, with many programs intentionally designed to cultivate leadership skills in participants. These experiences help individuals develop key leadership attributes, including effective communication, decision-making, conflict resolution, and the ability to inspire and motivate others [26]. Research indicates that experiential learning, such as that provided by AE, is particularly effective in fostering leadership because it allows participants to practice and reflect on their leadership behaviours in a real-world context [71].

Among the selected articles, four studies [57,63–65] indicated that AE programs foster the development of leadership skills among participants. Engaging in challenging outdoor activities requires individuals to take charge, make decisions, and guide their peers, leading to increased leadership abilities. The following quote testifies to that.

*“We had a couple people that weren’t really leaders, vocal leaders, but when it was their turn for leader of the day, they really had to step up and burst their comfort zone. And I think a lot of people should take advantage of that because that helps a lot. It boosts your confidence and you reassure yourself that you can be a leader”* [63].

The experiences that participants learnt to take on leadership roles are essential for developing important skills. These experiences not only improve leadership abilities but also greatly enhance personal responsibility and accountability. Such qualities are vital for success in both personal and professional areas. By engaging in these experiences, individuals learn to manage their own actions and navigate the complexities of leading others, ultimately preparing them for future challenges and opportunities.

#### Self-efficacy.

Self-efficacy refers to individuals’ beliefs regarding their capacity to achieve specific performance levels, influencing the events impacting their future lives.

These beliefs are pivotal in shaping individuals’ emotions, thoughts, motivation, and behaviour [72]. The analysis of five articles [44,57,58,63,65] demonstrated that AE enhances children’s self-efficacy by providing opportunities to overcome challenges and achieve goals in demanding environments. For instance, one participant reflected,

*“I’ve learned that if you really want to do something or really have to do something, you can actually do it even though you’re in bad conditions”* [65].

Such experiences develop a stronger belief in their capabilities by overcoming obstacles and accomplishing goals in adventurous settings. By accomplishing tasks in adventurous settings, participants get a deeper understanding of their own potential, which positively influences their motivation and readiness to face future challenges. This aligns with existing research that underscores the role of AE in enhancing self-efficacy among the participants [73].

#### Group belongings.

AE programs are structured around group activities to encourage participants to engage with one another and rely on each other’s support [74]. AE programs are intentionally designed around group-based activities to foster collaboration, mutual reliance, and social connectedness among participants [64] Additionally, the presence of supportive peers contributes to an increased sense of social connectedness and a feeling of belonging within the group [26]. The findings from multiple studies [57,58,64] emphasized the significance of group belonging as a critical outcome of AE, underscoring its role in socio-emotional development. Participants often reported an increased sense of social connectedness and belonging within their groups, which contributed to their emotional well-being. For instance, one participant reflected,

*“our group became more compassionate each day for each other, we care for each other,” and “[everybody was] making sure everyone’s safe”* [64].

These reflections highlight the supportive and empathetic environment cultivated through AE programs [26]. Activities like sail-training and outdoor expeditions demonstrate how participants often experience significant improvements in these areas, likely due to the strong sense of community and support that these programs foster [60]. This connection among participants can lead to increased motivation and personal growth, highlighting the importance of social dynamics in educational experiences outside the conventional classroom. These programs also help develop transferable groupwork skills, improve attitudes towards groupwork, and create a cooperative social environment [75].

In summary, the group-focused structure of AE programs creates a supportive and inclusive environment where participants can build meaningful connections, supporting their sense of belonging and contributing to their overall socio-emotional development. Structured activities, experiential learning, and reflective practices are essential mechanisms that foster this sense of belonging. This reinforces the transformative potential of AE in fostering not only individual growth but also a strong sense of community.

#### Stress relief.

Immersion in natural environments, combined with engaging in challenging and adventurous activities, provides participants with an opportunity to disconnect from the pressures of daily life and focus on the present moment. This combination fosters relaxation, rejuvenation, and an overall sense of emotional well-being [76] The thematic analysis also identified the role of AE in stress reduction, as noted by three articles [57,64,65]. Participants frequently reflected on the calming and restorative effects of being surrounded by nature. As one participant expressed,

*“Being in nature has also relieved a lot of stress and taken away some of my responsibilities”* [57].

The findings align with existing research that underscores the restorative impacts of natural environments on stress relief [77]. Exposure to nature has been shown to lower cortisol levels, reduce anxiety, and improve mood, which are critical components of stress relief [77]. Furthermore, the PA involved in AE contributes to the release of endorphins, which are known to enhance emotional well-being [78,79].

In essence, AE provides participants with a holistic approach to stress relief by combining the therapeutic effects of nature with the psychological benefits of physical and social engagement. This approach alleviates stress and equips individuals with healthier coping mechanisms, thereby enhancing their long-term emotional resilience and overall well-being.

#### Relationship skills.

AE programs play a significant role in enhancing participants’ relationship skills by providing opportunities to collaborate, mediate conflicts, and communicate effectively in challenging outdoor environments [80]. Findings from four studies [44,57,64,65] underscored the importance of AE in fostering positive interpersonal relationships by encouraging participants to work together, resolve disagreements, and build trust within their groups.

The structured nature of AE activities requires individuals to rely on one another for support, fostering mutual respect and understanding. One participant observed,

*“we don’t all sit together at recess and lunch but when we go on camp we all just become these cute little groups that just get along”* [65].

This sense of unity and camaraderie, developed through shared challenges and accomplishments, reflects the capacity of AE to break down social barriers and encourage meaningful connections.

By promoting collaboration, conflict resolution, and communication, AE builds positive interpersonal relationships and prepares participants for healthier social interactions in their everyday lives [81].

### Independence

AE fosters independence by encouraging participants to take initiative, make decisions, and navigate unfamiliar or challenging environments [82]. Findings from two studies [44,65] emphasized the role of AE in promoting independence among participants. By placing participants in situations where they must rely on their own judgment and abilities, AE helps cultivate a sense of ownership over their actions and decisions. Participants often highlighted the empowering nature of these experiences. One participant remarked,

*“like when they let us do something and don’t give us many rules… that’s pretty fun”* [65].

The emphasis on independence aligns with experiential learning theories that highlight the value of hands-on, self-directed learning [83]. AE settings create opportunities for participants to step out of their comfort zones, fostering confidence and resilience as they tackle challenges independently. Research has also shown that such experiences are critical for developing life skills, as they prepare individuals to handle real-world situations with greater competence and self-assurance [82]. In summary, AE serves as a powerful catalyst for promoting independence by challenging participants to take responsibility for their actions and decisions.

#### Following the guide.

The role of instructors and staff in AE programs is pivotal for fostering socio-emotional growth and creating a supportive environment for learning. Four studies [44,63–65] stressed the significance of the staff’s attitude and the role of instructors in AE programs. Positive and supportive staff members who provide guidance and ensure safety create a conducive environment for socio-emotional growth and learning. Participants highlighted the value of the guidance provided by instructors, with one noting:

*‘I think the instructors definitely helped us. A lot of the time they would help us think... to not [dwell] on the problems but think about the ways it can get better, the ways you can help yourself or others’* [63].

This statement reflects the instructors’ role in fostering problem-solving, resilience, and emotional regulation by encouraging constructive thinking and self-awareness. Skilled instructors not only ensure participants’ physical safety but also create a psychologically safe space where individuals feel supported to take risks, reflect on their experiences, and grow. Their ability to model positive behaviours, such as empathy, patience, and encouragement, further enhances participants’ interpersonal and intrapersonal development [84]. Knowledgeable and supportive instructors are essential to AE programs, as their guidance helps participants navigate challenges, reflect on their experiences, and cultivate practical and socio-emotional skills, fostering a transformative learning environment.

#### Theme 4: Connecting with nature.

One notable theme that was identified in the analysis is the impact of AE on the connection with nature. This theme encompasses two sub-themes: Connectedness with Wilderness and Spirituality.

#### Connectedness with wilderness.

AE plays a significant role in fostering a connection with nature, promoting environmental awareness, and encouraging positive behaviour changes [85]. AE fosters a profound connection between participants and the natural world by immersing them in wilderness settings such as forests, mountains, and rivers. AE unites conventional practices with modern practices to create stronger connections with the natural environment [86]. Three articles [58,65,66] emphasised the profound connection individuals experience with the wilderness through AE. Immersion in natural environments, such as forests, mountains, and rivers, allows participants to develop a deep appreciation for nature and a sense of belonging to the natural world. One described the connection vividly:

*‘[This experience is] Worth it. So worth it. [Because of] the beauty, and the good feeling that you have when you sit down in that cool mountain air when you’re covered in sweat from your hike. Cooling down and just feeling the breeze. Feeling the ground under you while you’re sitting. Tasting the air. When you dip your feet into the lake at the top of the mountain’* [58].

This reflection illustrates the sensory and emotional richness of engaging with wilderness, fostering a deep sense of mindfulness and presence.

#### Spirituality.

AE programs can provide participants with profound spiritual experiences, as highlighted in two studies [44,66]. Engaging in outdoor adventures can evoke a sense of fear, wonder, and connection to a higher power or spiritual dimension [87]. These experiences allow individuals to reflect on their place within the natural world and their relationship with something greater than themselves. Participants have shared moments of spiritual significance, such as one who reflected,

*‘Earlier, when I skinned a fish, I honoured it by burning its skin and thanking the creator for providing that fish for me’* [66].

This act of gratitude and reverence had demonstrated how AE can encourage participants to engage in meaningful rituals and connect with their spiritual beliefs through interactions with nature.

### Association of AE with physical, cognitive and socio-emotional development

Research findings from quantitative studies have revealed the extensive and varied developmental benefits that AE programs can offer to children (Table 2). These advantages encompass physical, cognitive, and socio-emotional aspects, highlighting the comprehensive influence of AE programmes on children’s overall development.

**Table 2:** Characteristics of selected quantitative studies.

Study	Study Design	Participant Characteristics	Intervention	Instrument and Data Collection	Result
Blaine and Akhurst (2020) South Africa	Quasi-experiment	S: *N = *144(males = 98, females = 54) Mean age = 16.5 years, SD = 3.45 years)	21 days Activities: canoeing, cycling, running, and hiking.	-Life Effectiveness Questionnaire, Emotional Literacy Questionnaire, and Connor-Davidson Resilience Scale were used -Data collected: pre- and post-Journey and four months later.	-Life effectiveness (*p* < 0.001, ηp^2 ^= 0.16) -Resilience (*p* = 0.01, ηp^2^ = 0.064) Emotional Literacy (*p* = 0.12, ηp^2^ = 0.03)
Chung et al. (2021) Hong Kong	Randomized Control Trial	S: *N* = 228; EG: 115, CG: 113 Male: 125, Female: 103 Age: 12–16 years (13.0 ± 0.8 years)	2-day/1-night Activities: spider web, toxic waste, abseiling, crossing the river using planks, wall climbing, high rope adventure, nocturnal hike, trampoline, and crossing a single-log bridge.	-Chinese version of the Center for Epidemiologic Studies Depression Scale for Children (CES-DC), The Chinese version of Rosenberg’s Self-Esteem Scale (RSES), and the 14-item Resilience Scale (RS-14) used -Data Collected: Baseline, 3 & 6 months of intervention	- Resilience (p = 0.01, ηp^2^ = 0.05) - Self-esteem (p = 0.01, ηp^2^ = 0.02) - Depressive symptoms(p = 0.01, ηp^2^ = 0.03)
Mackenzie et al. (2018) New-Zealand	One group experiment	N = 22 (41% females, 59% males)M = 15.7 years	− 05 days -Activities: Skiing	- Short Flow State Scale, Intrinsic Motivation Inventory, Learning Climate Questionnaire, Physical activity attitudes, Active outdoor identity and Pedometers were used -Data Collection: 1 month before, during, and 1 month after the intervention	-PA (p < .001, ηp^2^ = .663) -Intrinsic motivation (p < .001, ηp^2^ = .47) -Self-determination • Relatedness (p < .001, ηp^2^ = .44) • Competence (p < .001, ηp^2^ = .19) • Autonomy (p < .001, ηp^2^ = .52
Mutz et al. (2018) Germany	Longitudinal research design	N = 108 (M:48; F:28) Age: 13–20 (M = 17.8; SD = 1.26)	−10-days -Activities: canoeing, rappelling, cave expedition, canyoneering, rock climbing, fixed rope route and hiking tours.	-Perceived Stress Questionnaire, Life satisfaction question, and Hedonic balance were used. -Data collection: Pre and post-intervention	-High media consumers PSQ worry (p < 0.001) PSQ tension (p < 0.001) PSQ demand (p < 0.001) PSQ joy (p < 0.001) Hedonic balance (p < 0.001) Life satisfaction (p = .008) -Low-to-moderate media consumers PSQ worry (p = 0.001) PSQ tension (p = 0.022) PSQ demand (p < .001) PSQ joy (p = 0.015) Hedonic balance (p < .001) Life satisfaction (p = 0.538)
Mutz & Müller (2016) Germany	One group experiment	N = 12 (M: 7; F:5) Age: 14	− 09 days -Activities: Hiking	- Perceived Stress Questionnaire, General Self-efficacy Scale, Mindful Attention and Awareness Scale, and Subjective well-being questions were used -Data Collection: 1 week before and 4 days after the hike	-Perceived stress (p = .022, ES =−0.66) -Mindfulness (p = .001, ES = 1.32) -Life satisfaction (p = .034, ES = 0.58) -Self-efficacy (p = .188)
Scarf et al. (2017) New Zealand	Quasi-experiment	Study 1: N = 173 (M:72, F: 101) IG:100 (M:52, F:48); CG: 73 (M:20, F:53) Age: 15–19 years Study 2: N = 171 (M:58, F: 113) IG:80 (M:33, F:47); CG: 91 (M:25, F:66) Age: 15–19 years	−10 days -Activities: Voyage	-Life Effectiveness self-concept sub-scale, Sheldon and Bettencourt’s Group Belongings inclusion scale, and Self-Description Questionnaire III were used -Data Collection: First day and 10^th^ day	Study 1: -Group belonging (p < .001, η2p=0.242) -Self-esteem (p < .001) Study 2: -Self-esteem (T-1) r = 0.408, p < .001 -Self-esteem (T-2) r = 0.802, p < .001 -Self-efficacy (T-2) r = 0.337, p = 0.002 -Group belonging (T-2) r = 0.421, p < .001 - Group esteem (T-2) r = 0.304, p < .001

#### Impact of AE on physical development.

In the selected articles, only one study [62] measured participants’ PA levels using a pedometer. The other quantitative studies did not examine PA as an outcome. During the intervention, researchers found that the students’ average daily step count increased significantly, from 1,324 steps to 7,963 steps, representing a 121% increase. The students took 5,975–14,090 steps per day during the intervention, with all participants increasing their steps and over 90% exceeding their maximum baseline steps per day recorded in school. In fact, the mean steps per day during the intervention exceeded the general health recommendation of 10,000 steps per day [88]. However, the study found no significant differences in the students’ PA in pre- and post-school settings. The findings suggested AE increased participants’ PA levels during the intervention, but the lack of significant differences in PA between pre- and post-school settings indicated that the benefits may not have translated to overall lifestyle changes [89].

#### The impact of AE on cognitive development.

Only one study [62] assessed the learning environment of the participants by using the Learning Climate Questionnaire. The results suggest that the AE created a more positive and engaging learning environment than the regular school setting. During the programme, most students (90.5%) reported high learning climate scores of 5.5 or above on a 7-point scale, and none fell below 4. In contrast, the learning climate scores in the school settings were substantially lower, with the lowest scores being 1 (pre-course) and 2.1 (post-course). These findings indicate that the AE program fostered a significantly more positive and engaging learning environment in contrast to the traditional school setting.

#### Impact of AE on social and emotional development.

AE has positively impacted social and emotional development in several studies. Chung et al. [47] observed that participants in the experimental group exhibited statistically significant enhancements in resilience and reduced depressive symptoms compared to the placebo control group three and six months after the intervention. They have reported significant improvement in self-esteem in the experimental group at T-2 but not in T-3. Blaine and Akhurst [57] found that the intervention enhanced resilience among the participants and positively impacted the overall life effectiveness of the learners. However, they did not observe any improvement in emotional literacy. The study conducted by Mutz et al. [59,61] revealed that individuals with high screen usage exhibited significant changes in various dimensions of mental health (including worry, tension, demand, joy, hedonic balance, and life satisfaction) during T-1 and T-2. Conversely, individuals with low to moderate media usage demonstrated changes in perceived stress, while their life satisfaction remained constant. Mackenzie et al. [62] reported that participants’ intrinsic motivation significantly increased compared to pre- and post-school settings. Scarf et al. [60] found that the voyage program positively impacted self-esteem and group belonging among participants. Mutz and Müller [61] documented AE’s positive effects on participants’ mental well-being, including demand, mindfulness, and life satisfaction.

The existing research suggests that adventure education has diverse positive effects on the social-emotional well-being of participants. While individual impacts may vary across studies, long-term benefits can be observed by interventions with subsequent follow-ups.

## Discussion

The mixed-method systematic review strives to find AE programmes’ association with children’s physical, cognitive and social-emotional well-being. The first AE initiative was launched early in 1900, primarily focusing on human development for leisure purposes. Subsequently, it gained recognition in educational and therapeutic spheres due to its emphasis on immersing individuals in natural environments alongside their peers, thereby being perceived as a method to cultivate more responsible citizens [48]. Adventure education has gained immense popularity among educational institutions and tourists across the globe, catering to the needs of individuals, organisations, and movements. Its ability to offer a unique learning experience while being thrilling and adventurous has made it a sought-after activity for people looking to challenge themselves. However, the popularity of AE appears to be primarily confined to the Western world. The current review found only one study from Asia [47] and two from Africa [44,57]. Most of the research on this topic has been conducted in North America [58,63,64,66], Europe [59,61], and Oceania [60,62,65]. This suggests a significant lack of research on AE in low and middle-income countries, especially in Asia.

The World Health Organization identifies physical inactivity as the fourth leading risk factor for global mortality, with 1.8 billion adults are at risk of diseases due to insufficient physical activity [90]. The lack of PA or a completely sedentary lifestyle often leads to various health problems, such as postural issues [91], overweight and obesity [92,93], circulatory problems [94,95] and even premature death [96,97]. AE programs offer a promising approach to encouraging PA and holistic well-being among children and adolescents. The inherently challenging and hands-on nature of AE activities, such as rock climbing, hiking, and team-building exercises, motivates participants to push their physical boundaries in a supportive and adventurous environment. Pervious meta-analysis [98] indicated that PA programs have significant positive effects on non-executive (ES: 0.23), executive (ES: 0.20), and metacognitive (ES: 0.23) functions in children and adolescents. They reported that structured physical education and interventions to increase exercise levels were particularly effective [98]. The current review highlights that participants’ levels of PA were significantly higher during the AE intervention compared to their pre- and post-school settings [62]. Qualitative studies revealed that students recognised the physically demanding nature of the intervention, occasionally facing challenges in completing it. However, they demonstrated resilience by pushing themselves beyond their limits. that the current review found that youth encounter various physical obstacles during the intervention [64]. Males tend to be more inclined to embrace physically challenging activities to showcase strength and gain status [64]. At the same time, females were more affected by the loss of supportive relationships and prefer outdoor activities that foster intimate connections [44]. Future interventions should ensure equal benefits for all genders and avoid imposing excessive demands. Researchers found that AE programs helped children develop cognitive skills. By immersing students in unfamiliar environments, these programs enabled them to learn self-care skills. Additionally, AE improved academic performance compared to traditional classroom settings [99]. The AE programme enhanced participants’ critical thinking abilities [100] and improved their learning motivation [101,102], as well as their general problem-solving capabilities [33,103]. A one-year placement-based research project reported that 75% of students gained new knowledge about the subject matter through self-evaluation learning assessments [32]. The current review found similar beneficial outcomes from AE programs. Blaine and Akhurst [57] reported a positive effect on learners’ overall life effectiveness skills after participating in AE. The demanding AE activities also increased participants’ attention [58]. The active processes of engaging with specific challenges on the course led to their learning [64]. Additionally, AE experiences foster a connected academic community through shared stories of challenges and peak experiences [53]. Furthermore, Mackenzie et al. [62] reported improving student engagement in science education through AE intervention.

These findings highlight the importance of incorporating AE into educational curricula to support students’ cognitive growth. However, only one study has measured the learning outcomes of AE quantitatively. Future research should incorporate follow-up assessments after participating in AE activities to evaluate the improvement in cognitive development of these programs over time.

The role of social and emotional development in fostering critical life skills is fundamental. Social and emotional learning enables children to acquire and practice the skills necessary for academic success, fulfilling careers, healthy relationships, and responsible civic engagement (Widyananti, 2024 #201). Research has consistently demonstrated the positive outcomes of prioritising SEL in schools, including improved academic performance and decreased stress and anxiety [104]. Research has found that children participating in school-based AE programs exhibit better socio-emotional development [75,105]. These programs foster positive attitudes and mindsets that help participants overcome challenges, self-doubt, and distress. Similar findings have been found in this review. Both qualitative and quantitative research have documented the positive relationship between AE and the development of socio-emotional competencies.

For instance, study by Chung et al. [38] found a significant increase in resilience among participants after a 3-month AE intervention, although the effects were no longer significant at a 6-month follow-up. Similarly, Mutz and Muller [61] observed improvements in resilience during the intervention, but the long-term impact remains unclear due to the lack of prolonged follow-up. Participants in the study by Down et al. [65] echoed these findings too, expressing a sense of empowerment and accomplishment after tackling challenging outdoor situations.

Various challenging and risky activities, such as backpacking, sailing, and rafting, are inherent to AE programs, which require participants to work collaboratively with their peers. This collaborative engagement had increased participants’ self-esteem and self-efficacy, alongside a greater sense of group belonging [47,59,60]. Specifically, studies have reported a strong and unique predictive relationship between sail training and increased self-esteem [60] and moderate to large effect sizes for improvements in self-efficacy among AE participants [59]. Additionally, qualitative accounts from participants in this review studies have consistently supported the positive effects of AE interventions on enhancing self-efficacy and self-esteem, which in turn enable them to become more competent in problem-solving, coping with adverse situations, and building connections with known and unknown individuals [44,57,58,63,64]. These findings are further corroborated by a recent meta-analysis that reported a medium effect size of AE interventions on improving self-efficacy among participants [106].

In the modern job market, employers increasingly seek candidates who can make independent decisions, solve problems effectively, plan and organise tasks, and delegate work based on the abilities of their team members. However, the growing trend of nuclear families and the boom in technology have led many children to become confined within their own rooms, limiting their opportunities to take initiative and complete tasks independently. This, in turn, has resulted in a notable deficit in the communication and leadership skills that are crucial for securing employment in the future. The findings of the present review suggest that participation in AE programs can significantly bridge this gap by enhancing students’ leadership abilities. The structured environment provided by AE instructors encourages participants to step out of their comfort zones, leading to increased intrinsic motivation and a deeper understanding of the various facets of leadership, including communication and conflict resolution [62,64]. Qualitative accounts from participants further validate these findings, highlighting how the experience of leading their own groups during AE activities has fostered a sense of independence and autonomy [63]. However, Widmer et al. [107] noted that effective identity development through AE intervention requires a duration of at least two weeks. Among the 12 studies reviewed, three lasted over two weeks [44,57,64]. This suggests that the duration of AE intervention significantly impacts the outcomes. Subsequent research should explore extending the duration of their interventions.

Participating in AE intervention offers a refreshing break from academic pressure and daily routines, helping individuals steer clear of excessive media use. Notably, research by Mutz et al. [59] has found that individuals with high and moderate levels of media use tend to benefit the most from these AE experiences, experiencing greater gains compared to their peers with lower media consumption. This suggests that the AE environment can particularly impact those accustomed to high media engagement. Furthermore, participants in studies conducted by Blaine and Akhurst [57] have reported similar experiences of the benefits of a lack of technology and social media access during their AE programs, highlighting the positive impact on developing their social skills. This aligns with the broader finding that spending time in wilderness settings can reduce perceived stress [61] and depressive symptoms [47]. These findings underscore the value of AE interventions in providing a novel, technology-limited environment that can positively influence participants’ well-being and social development, particularly for those with high levels of media consumption in their daily lives.

### Strengths and limitations

The present study conducted a comprehensive review of recent literature to investigate the impact of AE on children’s physical, socio-emotional, and cognitive development. Both qualitative and quantitative measures were utilised to assess the effectiveness of AE interventions among children. However, it is important to acknowledge that the review is subject to certain limitations. The selected articles exhibited considerable heterogeneity regarding intervention types, duration, outcomes measured, and participants’ age, which pose challenges in drawing conclusive findings. Furthermore, while one study implemented a randomized controlled trial with single-blinded randomization, most studies lacked a comparison group and sufficient follow-up measures. It is worth noting that only 5 out of 12 articles (42%) were rated as high-quality based on the researchers’ quality appraisal. Additionally, the study exclusively focused on healthy children, necessitating a cautious interpretation of the results.

## Conclusion

This systematic review provides valuable insights into the effects of AE on children’s development. The programs have enhanced critical thinking, group belonging, resilience, self-esteem, academic efficacy and physical well-being. However, long-term effects could not be definitively concluded. Future research should include follow-up measures to assess sustained impact.

## Supporting information

Table S1 Studies identified in the literature search. (DOCX)

Table S2 List of full-text studies selected for the systematic review and the reasons for the exclusion of studies. (DOCX)

S3 PRISMA 2020 checklist PLOS One. (DOCX)

## References

[1] Dimitri P, Joshi K, Jones N, Moving Medicine for Children Working Group. Moving more: physical activity and its positive effects on long term conditions in children and young people. Arch Dis Child. 2020;105(11):1035–40. doi: 10.1136/archdischild-2019-318017 32198161

[2] Bull FC, Al-Ansari SS, Biddle S, Borodulin K, Buman MP, Cardon G, et al. World Health Organization 2020 guidelines on physical activity and sedentary behaviour. Br J Sports Med. 2020;54(24):1451–62. doi: 10.1136/bjsports-2020-102955 33239350 PMC7719906

[3] Caspersen CJ, Powell KE, Christenson GM. Physical activity, exercise, and physical fitness: definitions and distinctions for health-related research. Public health reports. 1985;100(2):126. 3920711 PMC1424733

[4] Warburton DER, Bredin SSD. Reflections on Physical Activity and Health: What Should We Recommend? Can J Cardiol. 2016;32(4):495–504. doi: 10.1016/j.cjca.2016.01.024 26995692

[5] Hinkley T, Brown H, Carson V, Teychenne M. Cross sectional associations of screen time and outdoor play with social skills in preschool children. PLoS One. 2018;13(4):e0193700. doi: 10.1371/journal.pone.0193700 29617366 PMC5884481

[6] Cattuzzo MT, Dos Santos Henrique R, Ré AHN, de Oliveira IS, Melo BM, de Sousa Moura M, et al. Motor competence and health related physical fitness in youth: A systematic review. J Sci Med Sport. 2016;19(2):123–9. doi: 10.1016/j.jsams.2014.12.004 25554655

[7] Skrede T, Steene-Johannessen J, Anderssen SA, Resaland GK, Ekelund U. The prospective association between objectively measured sedentary time, moderate-to-vigorous physical activity and cardiometabolic risk factors in youth: a systematic review and meta-analysis. Obes Rev. 2019;20(1):55–74. doi: 10.1111/obr.12758 30270500

[8] van Biljon A, McKune AJ, DuBose KD, Kolanisi U, Semple SJ. Do Short-Term Exercise Interventions Improve Cardiometabolic Risk Factors in Children? J Pediatr. 2018;203:325–9. doi: 10.1016/j.jpeds.2018.07.067 30172428

[9] Väistö J, Haapala EA, Viitasalo A, Schnurr TM, Kilpeläinen TO, Karjalainen P, et al. Longitudinal associations of physical activity and sedentary time with cardiometabolic risk factors in children. Scand J Med Sci Sports. 2019;29(1):113–23. doi: 10.1111/sms.13315 30276872 PMC6485341

[10] Pate RR, Hillman CH, Janz KF, Katzmarzyk PT, Powell KE, Torres A, et al. Physical Activity and Health in Children Younger than 6 Years: A Systematic Review. Med Sci Sports Exerc. 2019;51(6):1282–91. doi: 10.1249/MSS.0000000000001940 31095085 PMC6527328

[11] Erickson KI, Hillman CH, Kramer AF. Physical activity, brain, and cognition. Current Opinion in Behavioral Sciences. 2015;4:27–32.

[12] Kao S-C, Cadenas-Sanchez C, Shigeta TT, Walk AM, Chang Y-K, Pontifex MB, et al. A systematic review of physical activity and cardiorespiratory fitness on P3b. Psychophysiology. 2020;57(7):e13425. doi: 10.1111/psyp.13425 31228362

[13] Singh AS, Saliasi E, van den Berg V, Uijtdewilligen L, de Groot RHM, Jolles J, et al. Effects of physical activity interventions on cognitive and academic performance in children and adolescents: a novel combination of a systematic review and recommendations from an expert panel. Br J Sports Med. 2019;53(10):640–7. doi: 10.1136/bjsports-2017-098136 30061304

[14] Wu C-T, Hillman CH. Aerobic fitness and the attentional blink in preadolescent children. Neuropsychology. 2013;27(6):642–53. doi: 10.1037/a0034025 24059445

[15] Zeng N, Ayyub M, Sun H, Wen X, Xiang P, Gao Z. Effects of Physical Activity on Motor Skills and Cognitive Development in Early Childhood: A Systematic Review. Biomed Res Int. 2017;2017:2760716. doi: 10.1155/2017/2760716 29387718 PMC5745693

[16] Chekroud SR, Gueorguieva R, Zheutlin AB, Paulus M, Krumholz HM, Krystal JH, et al. Association between physical exercise and mental health in 1·2 million individuals in the USA between 2011 and 2015: a cross-sectional study. Lancet Psychiatry. 2018;5(9):739–46. doi: 10.1016/S2215-0366(18)30227-X 30099000

[17] Rodriguez-Ayllon M, Cadenas-Sánchez C, Estévez-López F, Muñoz NE, Mora-Gonzalez J, Migueles JH, et al. Role of Physical Activity and Sedentary Behavior in the Mental Health of Preschoolers, Children and Adolescents: A Systematic Review and Meta-Analysis. Sports Med. 2019;49(9):1383–410. doi: 10.1007/s40279-019-01099-5 30993594

[18] White RL, Babic MJ, Parker PD, Lubans DR, Astell-Burt T, Lonsdale C. Domain-Specific Physical Activity and Mental Health: A Meta-analysis. Am J Prev Med. 2017;52(5):653–66. doi: 10.1016/j.amepre.2016.12.008 28153647

[19] Carson V, Tremblay MS, Chaput J-P, McGregor D, Chastin S. Compositional analyses of the associations between sedentary time, different intensities of physical activity, and cardiometabolic biomarkers among children and youth from the United States. PLoS One. 2019;14(7):e0220009. doi: 10.1371/journal.pone.0220009 31329609 PMC6645531

[20] Katzmarzyk PT, Barreira TV, Broyles ST, Champagne CM, Chaput J-P, Fogelholm M, et al. Physical Activity, Sedentary Time, and Obesity in an International Sample of Children. Med Sci Sports Exerc. 2015;47(10):2062–9. doi: 10.1249/MSS.0000000000000649 25751770

[21] World Health Organization. WHO guidelines on physical activity and sedentary behaviour. 2020 [cited 2025 19.05]. https://www.who.int/publications/i/item/9789240015128 33369898

[22] Barrable A, Booth D. Nature Connection in Early Childhood: A Quantitative Cross-Sectional Study. Sustainability. 2020;12(1):375. doi: 10.3390/su12010375

[23] Clements R. An Investigation of the Status of Outdoor Play. Contemporary Issues in Early Childhood. 2004;5(1):68–80. doi: 10.2304/ciec.2004.5.1.10

[24] Koszałka-Silska A, Korcz A, Wiza A. The impact of physical education based on the adventure education programme on self-esteem and social competences of adolescent boys. International Journal of Environmental Research and Public Health. 2021;18(6):3021. 33804140 10.3390/ijerph18063021 PMC8000378

[25] Lee J, Zhang T. The impact of adventure education on students’ learning outcomes in physical education: A systematic review. JTRM in Kinesiology. 2019.

[26] Hutson G, Baird J, Ives CD, Dale G, Holzer JM, Plummer R. Outdoor adventure education as a platform for developing environmental leadership. People and Nature. 2024;6(5):1974–86. doi: 10.1002/pan3.10699

[27] Newton M, Sandberg J, Watson DL. Utilizing Adventure Education Within the Model of Moral Action. Quest. 2001;53(4):483–94. doi: 10.1080/00336297.2001.10491760

[28] Li X. Middle school student’s leadership skills and their PE class participation. J Educ Educ Res. 2024.

[29] Ganea V, Development Of Social Abilities In School Through Adventure Education; 2020.

[30] Davidson C, Ewert A, Chang Y. Multiple Methods for Identifying Outcomes of a High Challenge Adventure Activity. Journal of Experiential Education. 2016;39(2):164–78. doi: 10.1177/1053825916634116

[31] Fűz N. Out-of-School Learning in Hungarian Primary Education: Practice and Barriers. Journal of Experiential Education. 2018;41(3):277–94. doi: 10.1177/1053825918758342

[32] (Athman) Ernst * J, Monroe M. The effects of environment‐based education on students’ critical thinking skills and disposition toward critical thinking. Environmental Education Research. 2004;10(4):507–22. doi: 10.1080/1350462042000291038

[33] Hattie J, Marsh HW, Neill JT, Richards GE. Adventure Education and Outward Bound: Out-of-Class Experiences That Make a Lasting Difference. Review of Educational Research. 1997;67(1):43. doi: 10.2307/1170619

[34] Sibthorp J, Paisley K, Gookin J. Exploring Participant Development Through Adventure-Based Programming: A Model from the National Outdoor Leadership School. Leisure Sciences. 2007;29(1):1–18. doi: 10.1080/01490400600851346

[35] Sibthorp J, Furman N, Paisley K, Gookin J, Schumann S. Mechanisms of Learning Transfer in Adventure Education: Qualitative Results From the NOLS Transfer Survey. Journal of Experiential Education. 2011;34(2):109–26. doi: 10.5193/jee34.2.109

[36] Ewert A, Yoshino A. The influence of short-term adventure-based experiences on levels of resilience. Journal of Adventure Education & Outdoor Learning. 2011;11(1):35–50. doi: 10.1080/14729679.2010.532986

[37] Neill JT, Dias KL. Adventure education and resilience: The double-edged sword. Journal of Adventure Education & Outdoor Learning. 2001;1(2):35–42. doi: 10.1080/14729670185200061

[38] Bailey AW, Kang HK. Modeling the impact of wilderness orientation programs on first-year academic success and life purpose. Journal of Adventure Education and Outdoor Learning. 2015;15(3):209–23.

[39] Pirchio S, et al. The effects of contact with nature during outdoor environmental education on students’ wellbeing, connectedness to nature and pro-sociality. Frontiers in Psychology. 2021;12:648458. 34017288 10.3389/fpsyg.2021.648458 PMC8129515

[40] Wilson JD, Burnor AC. Association between an adventure education apprenticeship and at-risk urban youths’ resilience. World Leisure Journal. 2011;53(4):255–68. doi: 10.1080/04419057.2011.630784

[41] Chan HH-K, Kwong HYC, Shu GLF, Ting CY, Lai FH-Y. Effects of Experiential Learning Programmes on Adolescent Prosocial Behaviour, Empathy, and Subjective Well-being: A Systematic Review and Meta-Analysis. Front Psychol. 2021;12:709699. doi: 10.3389/fpsyg.2021.709699 34421761 PMC8371439

[42] Passarelli A, Hall E, Anderson M. A Strengths-Based Approach to Outdoor and Adventure Education: Possibilities for Personal Growth. Journal of Experiential Education. 2010;33(2):120–35. doi: 10.5193/jee33.2.120

[43] Deane KL, Harré N. The Youth Adventure Programming Model. J of Research on Adolesc. 2013;24(2):293–308. doi: 10.1111/jora.12069

[44] Zygmont CS, Naidoo AV. A phenomenographic study of factors leading to variation in the experience of a school-based wilderness experiential programme. South African Journal of Psychology. 2017;48(1):129–41. doi: 10.1177/0081246317690686

[45] Albedry B, Ammons L, Marenus MW, Hammoud D, Jandali D, Chrzanowski M, et al. The Effects of an Adventure Education Pilot Study on Social Emotional Learning, Resilience, and Physical Activity among High School Students. American Journal of Health Education. 2023;54(5):329–42. doi: 10.1080/19325037.2023.2234976

[46] Allan JF, Doran A, Jones R, Farrell S. Building resilience and well-being for post-covid adolescents through outdoor adventure. Journal of Adventure Education and Outdoor Learning. 2024;25(1):166–92. doi: 10.1080/14729679.2024.2312920

[47] Chung JOK, Li WHC, Ho KY, Lam KKW, Cheung AT, Ho LLK, et al. Adventure-based training to enhance resilience and reduce depressive symptoms among juveniles: A randomized controlled trial. Res Nurs Health. 2021;44(3):438–48. doi: 10.1002/nur.22127 33754400

[48] Gargano V, Turcotte D. Helping factors in an outdoor adventure program. Journal of Social Work. 2019;21(1):88–106. doi: 10.1177/1468017319863708

[49] Peng Z, Lau PWC. Effectiveness of Adventure Education on Health Outcomes Related to Physical, Psychological, and Social Development in Children: A Systematic Review. Journal of Teaching in Physical Education. 2023;42(3):511–24. doi: 10.1123/jtpe.2022-0106

[50] Shamseer L, Moher D, Clarke M, Ghersi D, Liberati A, Petticrew M, et al. Preferred reporting items for systematic review and meta-analysis protocols (PRISMA-P) 2015: elaboration and explanation. BMJ. 2015;350:g7647. doi: 10.1136/bmj.g7647 25555855

[51] Higgins J. Cochrane handbook for systematic reviews of interventions. Cochrane Collaboration and John Wiley & Sons Ltd; 2008.

[52] World Health Organization. Constitution. 1948 [cited 2025 19.05]. https://www.who.int/about/governance/constitution

[53] Lockwood C, Munn Z, Porritt K. Qualitative research synthesis: methodological guidance for systematic reviewers utilizing meta-aggregation. JBI Evidence Implementation. 2015;13(3):179–87. 10.1097/XEB.0000000000000062 26262565

[54] Barker TH, Stone JC, Sears K, Klugar M, Tufanaru C, Leonardi-Bee J, et al. The revised JBI critical appraisal tool for the assessment of risk of bias for randomized controlled trials. JBI Evid Synth. 2023;21(3):494–506. doi: 10.11124/JBIES-22-00430 36727247

[55] Higginbottom GMA, Hadziabdic E, Yohani S, Paton P. Immigrant women’s experience of maternity services in Canada: a meta-ethnography. Midwifery. 2014;30(5):544–59. doi: 10.1016/j.midw.2013.06.004 23948185

[56] Kabir MR, Chan K. Menopausal experiences of women of Chinese ethnicity: A meta-ethnography. PLoS One. 2023;18(9):e0289322. doi: 10.1371/journal.pone.0289322 37703245 PMC10499211

[57] Blaine J, Akhurst J. A South African exploration into outdoor adventure education and adolescent psychosocial development. Journal of Psychology in Africa. 2020;30(5):440–50. doi: 10.1080/14330237.2020.1821311

[58] Ingman BC. Adventure education as aesthetic experience. Journal of Adventure Education and Outdoor Learning. 2018;18(4):323–37. doi: 10.1080/14729679.2018.1470015

[59] Mutz M, Müller J, Göring A. Outdoor adventures and adolescents’ mental health: Daily screen time as a moderator of changes. Journal of Adventure Education and Outdoor Learning. 2019;19(1):56–66.

[60] Scarf D, Kafka S, Hayhurst J, Jang K, Boyes M, Thomson R, et al. Satisfying psychological needs on the high seas: explaining increases self-esteem following an Adventure Education Programme. Journal of Adventure Education and Outdoor Learning. 2017;18(2):165–75. doi: 10.1080/14729679.2017.1385496

[61] Mutz M, Müller J. Mental health benefits of outdoor adventures: Results from two pilot studies. J Adolesc. 2016;49:105–14. doi: 10.1016/j.adolescence.2016.03.009 27038974

[62] Mackenzie SH, Son JS, Eitel K. Using outdoor adventure to enhance intrinsic motivation and engagement in science and physical activity: An exploratory study. Journal of Outdoor Recreation and Tourism. 2018;21:76–86. doi: 10.1016/j.jort.2018.01.008

[63] Richmond D, Sibthorp J, Gookin J, Annarella S, Ferri S. Complementing classroom learning through outdoor adventure education: out-of-school-time experiences that make a difference. Journal of Adventure Education and Outdoor Learning. 2017;18(1):36–52. doi: 10.1080/14729679.2017.1324313

[64] Orson CN, McGovern G, Larson RW. How challenges and peers contribute to social-emotional learning in outdoor adventure education programs. Journal of Adolescence. 2020;81:7–18. 32247894 10.1016/j.adolescence.2020.02.014

[65] Down M, Picknoll D, Piggott B, Hoyne G, Bulsara C. “I love being in the outdoors”: A qualitative descriptive study of outdoor adventure education program components for adolescent wellbeing. J Adolesc. 2023;95(6):1232–44. doi: 10.1002/jad.12197 37226929

[66] Ritchie SD, Wabano MJ, Corbiere RG, Restoule BM, Russell KC, Young NL. Connecting to the Good Life through outdoor adventure leadership experiences designed for Indigenous youth. Journal of Adventure Education and Outdoor Learning. 2015;15(4):350–70. doi: 10.1080/14729679.2015.1036455

[67] Campbell R, et al. Evaluating meta ethnography: systematic analysis and synthesis of qualitative research; 2012. 10.3310/hta15430 22176717

[68] Peacock J, Bowling A, Finn K, McInnis K. Use of Outdoor Education to Increase Physical Activity and Science Learning among Low-Income Children from Urban Schools. American Journal of Health Education. 2021;52(2):92–100. doi: 10.1080/19325037.2021.1877222

[69] Towner B, Cybulski S, Bolick B. Enhancing physical activity through outdoor activities: Strategies for incorporating nature during the school day. Journal of the Appalachian Institute for Health and Wellness. 2024.

[70] James JK, Williams T. School-Based Experiential Outdoor Education. Journal of Experiential Education. 2017;40(1):58–71. doi: 10.1177/1053825916676190

[71] Priest S. Mechanisms of change for adventure: four pathways through the “black box” process. Journal of Outdoor and Environmental Education. 2023;27(2):309–20. doi: 10.1007/s42322-023-00126-4

[72] Bandura A, Wessels S. Self-efficacy. Cambridge: Cambridge University Press; 1997.

[73] Hovey K, Niland D, Foley JT. The Impact of Participation in an Outdoor Education Program on Physical Education Teacher Education Student Self-Efficacy to Teach Outdoor Education. Journal of Teaching in Physical Education. 2020;39(1):18–27. doi: 10.1123/jtpe.2018-0288

[74] Jostad J, et al. The adolescent social group in outdoor adventure education: social connections that matter. Research in Outdoor Education. 2015;13(1):16–37.

[75] Cooley SJ, Burns VE, Cumming J. Using Outdoor Adventure Education to Develop Students’ Groupwork Skills. Journal of Experiential Education. 2016;39(4):329–54. doi: 10.1177/1053825916668899

[76] Zhou P, Lau PW. The impact of adventure education on psychosocial well-being in adolescents: A systematic review. International Journal of Physical Education. 2022;59(2):2–16.

[77] Li WHC, Chung JOK, Ho EKY. Effectiveness of an adventure-based training programme in promoting the psychological well-being of primary schoolchildren. J Health Psychol. 2013;18(11):1478–92. doi: 10.1177/1359105312465102 23221616

[78] Chen C, Nakagawa S. Recent advances in the study of the neurobiological mechanisms behind the effects of physical activity on mood, resilience and emotional disorders. Adv Clin Exp Med. 2023;32(9):937–42. doi: 10.17219/acem/171565 37665079

[79] Dettweiler U, Gerchen M, Mall C, Simon P, Kirsch P. Choice matters: Pupils’ stress regulation, brain development and brain function in an outdoor education project. Br J Educ Psychol. 2023;93(Suppl 1):152–73. doi: 10.1111/bjep.12528 35872620

[80] Forgan JW, Jones CD. How Experiential Adventure Activities Can Improve Students’ Social Skills. TEACHING Exceptional Children. 2002;34(3):52–8. doi: 10.1177/004005990203400307

[81] Stuhr PT, et al. The road less traveled in elementary physical education: Exploring human relationship skills in adventure‐based learning. Education Research International. 2018;2018(1):3947046.

[82] Riley M, Sibthorp J, Rochelle S. The developmental value of independent student expeditions in outdoor adventure education for emerging adult-aged participants. Journal of Outdoor Recreation, Education, and Leadership. 2024;16(2).

[83] Kolb DA, Boyatzis RE, Mainemelis C. Experiential learning theory: Previous research and new directions. Perspectives on thinking, learning, and cognitive styles. Routledge. 2014. p. 227–47.

[84] Schumann SA, Paisley K, Sibthorp J, Gookin J. Instructor Influences on Student Learning at NOLS. Journal of Outdoor Recreation, Education, and Leadership. 2009;1(1). doi: 10.7768/1948-5123.1015

[85] García-González E, Schenetti M. Education in nature and learning science in early childhood: a fertile and sustainable symbiosis. Journal of Outdoor and Environmental Education. 2022;25(3):363–77. doi: 10.1007/s42322-022-00110-4

[86] Mitra S, Sharma-Brymer V, Mitten D, Ady J. India’s emerging trends and meanings in healthy human-nature relationships: Indian outdoor education practitioner perspectives. Journal of Adventure Education and Outdoor Learning. 2024;25(1):138–51. doi: 10.1080/14729679.2024.2350973

[87] Griffin J, LeDuc J. Out of the fish tank: The impact of adventure programs as a catalyst for spiritual growth. Leisure/Loisir. 2009;33(1):197–215. doi: 10.1080/14927713.2009.9651436

[88] Parra Saldías M, et al. How many daily steps are really enough for adolescents: a cross-validation study. 2018.

[89] Bullock SH, Jones BH, Gilchrist J, Marshall SW. Prevention of physical training-related injuries recommendations for the military and other active populations based on expedited systematic reviews. Am J Prev Med. 2010;38(1 Suppl):S156-81. doi: 10.1016/j.amepre.2009.10.023 20117590

[90] World Health Organization. Nearly 1.8 billion adults at risk of disease from not doing enough physical activity. 2024 [cited 2025 19.05]. https://www.who.int/news/item/26-06-2024-nearly-1.8-billion-adults-at-risk-of-disease-from-not-doing-enough-physical-activity PMC11288492 39074895

[91] Salsali M, Sheikhhoseini R, Sayyadi P, Hides JA, Dadfar M, Piri H. Association between physical activity and body posture: a systematic review and meta-analysis. BMC Public Health. 2023;23(1):1670. doi: 10.1186/s12889-023-16617-4 37649076 PMC10470156

[92] Bleich SN, Vercammen KA, Zatz LY, Frelier JM, Ebbeling CB, Peeters A. Interventions to prevent global childhood overweight and obesity: a systematic review. Lancet Diabetes Endocrinol. 2018;6(4):332–46. doi: 10.1016/S2213-8587(17)30358-3 29066096

[93] Morano M, Robazza C, Bortoli L, Rutigliano I, Ruiz MC, Campanozzi A. Physical Activity and Physical Competence in Overweight and Obese Children: An Intervention Study. Int J Environ Res Public Health. 2020;17(17):6370. doi: 10.3390/ijerph17176370 32883044 PMC7504542

[94] Falkner B, Lurbe E. Primordial Prevention of High Blood Pressure in Childhood: An Opportunity Not to be Missed. Hypertension. 2020;75(5):1142–50. doi: 10.1161/HYPERTENSIONAHA.119.14059 32223379

[95] Mora-Gonzalez J, Esteban-Cornejo I, Solis-Urra P, Migueles JH, Cadenas-Sanchez C, Molina-Garcia P, et al. Fitness, physical activity, sedentary time, inhibitory control, and neuroelectric activity in children with overweight or obesity: The ActiveBrains project. Psychophysiology. 2020;57(6):e13579. doi: 10.1111/psyp.13579 32249933

[96] Dempsey PC, Friedenreich CM, Leitzmann MF, Buman MP, Lambert E, Willumsen J, et al. Global Public Health Guidelines on Physical Activity and Sedentary Behavior for People Living With Chronic Conditions: A Call to Action. J Phys Act Health. 2021;18(1):76–85. doi: 10.1123/jpah.2020-0525 33276323

[97] Ekblom-Bak E, Halldin M, Vikström M, Stenling A, Gigante B, de Faire U, et al. Physical activity attenuates cardiovascular risk and mortality in men and women with and without the metabolic syndrome - a 20-year follow-up of a population-based cohort of 60-year-olds. Eur J Prev Cardiol. 2021;28(12):1376–85. doi: 10.1177/2047487320916596 34647588

[98] Alvarez-Bueno C, et al. The effect of physical activity interventions on children’s cognition and metacognition: A systematic review and meta-analysis. Journal of the American Academy of Child & Adolescent Psychiatry. 2017;56(9):729–38. 28838577 10.1016/j.jaac.2017.06.012

[99] Becker C, Lauterbach G, Spengler S, Dettweiler U, Mess F. Effects of Regular Classes in Outdoor Education Settings: A Systematic Review on Students’ Learning, Social and Health Dimensions. Int J Environ Res Public Health. 2017;14(5):485. doi: 10.3390/ijerph14050485 28475167 PMC5451936

[100] Setambah MAB. Adventure Learning in Basics Statistics: Impact on Students Critical Thinking. International Journal of Instruction. 2019;12(3):151–66.

[101] Bowker R, Tearle P. Gardening as a learning environment: A study of children’s perceptions and understanding of school gardens as part of an international project. Learning Environ Res. 2007;10(2):83–100. doi: 10.1007/s10984-007-9025-0

[102] Wistoft K. The desire to learn as a kind of love: gardening, cooking, and passion in outdoor education. Journal of Adventure Education & Outdoor Learning. 2013;13(2):125–41. doi: 10.1080/14729679.2012.738011

[103] Cason D, Gillis“Lee” HL. A Meta-Analysis of Outdoor Adventure Programming with Adolescents. Journal of Experiential Education. 1994;17(1):40–7. doi: 10.1177/105382599401700109

[104] Collaborative for Academic, S., and Emotional Learning (CASEL). Fundamentals of SEL; 2024 [cited 2025 20.05]. https://casel.org/fundamentals-of-sel/

[105] White RM. Adventure Based Learning Experience (ABLE). Journal of the American Academy of Special Education Professionals. 2007;75:86.

[106] Fang B-B, Lu FJH, Gill DL, Liu SH, Chyi T, Chen B. A Systematic Review and Meta-Analysis of the Effects of Outdoor Education Programs on Adolescents’ Self-Efficacy. Percept Mot Skills. 2021;128(5):1932–58. doi: 10.1177/00315125211022709 34107802

[107] Widmer MA, Duerden MD, Taniguchi ST. Increasing and generalizing self-efficacy: The effects of adventure recreation on the academic efficacy of early adolescents. Journal of Leisure Research. 2014;46(2):165–83.

