# Supplementary material for: Investigating the impact of adventure education on children’s physical, cognitive and socio-emotional development: A mixed method systematic review
Source: PLoS One. 2025 Jun 30;20(6):e0327181. doi: 10.1371/journal.pone.0327181 (PMC12208478; doi:10.1371/journal.pone.0327181)
Supplement: Table S1 — (DOCX) [file pone.0327181.s001.docx]

**Title:**

**Investigating the Impact of Adventure Education on Children’s Physical, Cognitive and Socio-emotional Development: A Mixed Method Systematic Review**

**Table S1: Studies Identified in the Literature Search**

| **Database** | **Search ID#** | **Search Terms** | **Search Options** | **Actions** | **Data Extractor(s) & Date** |
| --- | --- | --- | --- | --- | --- |
| **Sportdiscuss (EBSCOHost)** | S1 | ((adventure education OR adventure-based training OR outdoor adventure OR adventure experience OR adventure tourism OR adventure activity OR experiential learning OR experiential education) ) AND ( (Pre-schooler* OR Schoolchild* OR School-age OR Child* OR Pediatric* OR Adolescent* OR Youngster* OR Teen* OR Minor* OR Youth OR “Young person” OR Juvenile*) ) | **Limiters** - Publication Date: 20000101-20240131; Language: English  **Expanders** - Apply related words; Apply equivalent subjects  **Search modes** - Boolean/Phrase | 397 | RG, LK & 06/03/2024 |
| **Pubmed** | S1 | ((adventure education OR adventure-based training OR outdoor adventure OR adventure experience OR adventure tourism OR adventure activity OR experiential learning OR experiential education)) AND ((Pre-schooler* OR Schoolchild* OR School-age OR Child* OR Pediatric* OR Adolescent* OR Youngster* OR Teen* OR Minor* OR Youth OR "Young person" OR Juvenile*)) | **Limiters** - Meta-Analysis, Randomized Controlled Trial, Review, Systematic Review, from 2000 - 2024 | 404 | RG, LK & 06/03/2024 |
| **Medline** | S1 | (adventure education OR adventure-based training OR outdoor adventure OR adventure experience OR adventure tourism OR adventure activity OR experiential learning OR experiential education) AND (Pre-schooler* OR Schoolchild* OR School-age OR Child* OR Pediatric* OR Adolescent* OR Youngster* OR Teen* OR Minor* OR Youth OR "Young person" OR Juvenile*) {No Related Terms} |  | 58 | RG, LK & 06/03/2024 |
|  | S2 |  | limit 1 to (english language and humans and yr="2000 -Current") | 56 |  |
| **PsycINFO** | S1 | (adventure education OR adventure-based training OR outdoor adventure OR adventure experience OR adventure tourism OR adventure activity OR experiential learning OR experiential education) AND (Pre-schooler* OR Schoolchild* OR School-age OR Child* OR Pediatric* OR Adolescent* OR Youngster* OR Teen* OR Minor* OR Youth OR "Young person" OR Juvenile*) | Date: After January 01 2000 | 1062 | RG, LK & 06/03/2024 |
|  | S2 |  | Date: After January 01 2000  Methodology  Empirical Study, Experimental Replication, Field Study, Focus Group, Followup Study, Literature Review, Longitudinal Study, Meta Analysis, Nonclinical Case Study, Prospective Study, Qualitative Study, Quantitative Study, Retrospective Study, Systematic Review  Language  English | 1059 |  |
| **EMBASE (Ovid)** | S1 | (adventure education OR adventure-based training OR outdoor adventure OR adventure experience OR adventure tourism OR adventure activity OR experiential learning OR experiential education) AND (Pre-schooler* OR Schoolchild* OR School-age OR Child* OR Pediatric* OR Adolescent* OR Youngster* OR Teen* OR Minor* OR Youth OR "Young person" OR Juvenile*) {No Related Terms} |  | 385 | RG, LK & 06/03/2024 |
|  | S2 |  | limit 1 to (human and english language and yr="2000 -Current" and (children - focussed or "humans only (removes records about animals)")) | 360 |  |
| **Web of Science** | S1 | **(adventure education OR adventure-based training OR outdoor adventure OR adventure experience OR adventure tourism OR adventure activity OR experiential learning OR experiential education) AND (Pre-schooler* OR Schoolchild* OR School-age OR Child* OR Pediatric* OR Adolescent* OR Youngster* OR Teen* OR Minor* OR Youth OR “Young person” OR Juvenile*) (All Fields) and Article or Review Article or Early Access (Document Types) and English (Languages) and Public Environmental Occupational Health or Education Educational Research or Pediatrics or Environmental Sciences Ecology or Nutrition Dietetics or Psychology or Sport Sciences or Psychiatry or Science Technology Other Topics or Engineering or Computer Science or Social Sciences Other Topics or Behavioral Sciences or Food Science Technology or Urban Studies or Architecture or Women S Studies or Family Studies or Linguistics (Research Areas) and ANNUAL MEETING OF THE AMERICAN EDUCATIONAL RESEARCH ASSOCIATION or 17TH ANNUAL SCIENTIFIC MEETING OF ARCTICNET ASM or 3RD ANNUAL INTERNATIONAL WOKSHOP ON EMERGENCE AND EVOLUTION OF LINGUISTIC COMMUNICATION or 3RD INTERNATIONAL CONFERENCE ON ENTERTAINMENT COMPUTING ICEC 2004 or 44RD ANNUAL MEETING OF THE CANADIAN ASSOCIATION OF PAEDIATRIC SURGEONS CAPS or 4TH INTERNATIONAL CONFERENCE IN HOLISTIC HEALTH or 64TH INTERNATIONAL ASTRONAUTICAL CONGRESS IAC or ANNUAL MEETING OF THE ROYAL COLLEGE OF NURSING CHILD DEVELOPMENT AND DISABILITY GROUP or CLIVE L DYM MUDD DESIGN WORKSHOP X DESIGN AND THE FUTURE OF THE ENGINEER OF 2020 or CONFERENCE ON NEUROSCIENCES AND MUSIC MUTUAL INTERACTIONS AND IMPLICATIONS ON DEVELOPMENTAL FUNCTIONS (Exclude – Conference Titles)** |  | 705 | RG, LK & 06/03/2024 |
| **ERIC** | S1 | (adventure education OR adventure-based training OR outdoor adventure OR adventure experience OR adventure tourism OR adventure activity OR experiential learning OR experiential education) AND (Pre-schooler* OR Schoolchild* OR School-age OR Child* OR Pediatric* OR Adolescent* OR Youngster* OR Teen* OR Minor* OR Youth OR “Young person” OR Juvenile*) | **Limiters** - Publication Date: 20000101-20240131; Journal or Document: Journal Article (EJ); Education Level: Early Childhood Education, Elementary Education, Elementary Secondary Education, Grade 1, Grade 2, Grade 3, Grade 4, Grade 5, Grade 6, Grade 7, Grade 8, Grade 9, Grade 10, High Schools, Junior High Schools, Kindergarten, Preschool Education, Primary Education, Secondary Education; Intended Audience: Administrators, Community, Parents, Policymakers, Practitioners, Researchers, Students, Teachers; Language: English  **Expanders** - Apply equivalent subjects  **Search modes** - Boolean/Phrase | 66 |  |

In total: 3048

Duplicate: 441

After removing duplicates: 2813

Relevant full-text articles: 29

Selected for Review: 12
