# Supplementary material for: Investigating the impact of adventure education on children’s physical, cognitive and socio-emotional development: A mixed method systematic review
Source: PLoS One. 2025 Jun 30;20(6):e0327181. doi: 10.1371/journal.pone.0327181 (PMC12208478; doi:10.1371/journal.pone.0327181)
Supplement: Table S2 — (DOCX) [file pone.0327181.s002.docx]

| **Table S2: List of full-text studies selected for the systematic review and the reasons for the exclusion of studies** | | | |
| --- | --- | --- | --- |
| **First Author** | **Year of publication** | **Eligibility** | |
| Zygmont, C. S | 2017 | | Included |
| Judith Blaine | 2020 | | Included |
| Jessie Marguerite Barrie | 2005 | | Excluded: Dissertation |
| Joyce O. K. Chung | 2021 | | Included |
| Benjamin C. Ingman | 2018 | | Included |
| Gail Rizzo | 2016 | | Excluded: Dissertation |
| Sally Owens Palmer | 2015 | | Excluded: Dissertation |
| Tonje M. Molyneux | 2022 | | Excluded: Not conducted among adolescents |
| Virginie Gargano | 2021 | | Excluded: Not conducted among adolescents |
| James W. Forgan | 2002 | | Excluded: Report |
| Mitchell J. Fritz | 2022 | | Excluded: Dissertation |
| Andrew C. Grocott | 2009 | | Excluded: Not Adventure Education |
| Helker, K | 2022 | | Excluded: Focused only on challenges |
| Ingman, B. C. | 2018 | | Included |
| Mackenzie, S. H. | 2018 | | Included |
| Molyneux, T. M | 2023 | | Excluded: Not Adventure Education |
| Morgan, A | 2021 | | Excluded: Dissertation |
| Mutz, M. | 2016 | | Included |
| Mutz, M. | 2019 | | Included |
| Opper, B. | 2016 | | Excluded: Dissertation |
| Orson, C. N. | 2020 | | Included |
| Overholt, J. R. | 2022 | | Excluded: Not Adventure Education |
| Palmer, S. O. | 2016 | | Excluded: Dissertation |
| Prince, H. E. | 2021 | | Excluded: Systematic Review |
| Richmond, D. | 2018 | | Included |
| Richmond, D. J. | 2017 | | Excluded: Dissertation |
| Ritchie, S. D. | 2015 | | Included |
| Rizzo, G. | 2018 | | Excluded: Dissertation |
| Scarf, D. | 2018 | | Included |
| Zmudy, M. H | 2009 | | Excluded: Not Adventure Education |
